# Design, optimization, and comparative analysis of wide-band polarization conversion along with dual coding sequences for RCS reduction

**DOI:** 10.1038/s41598-024-59054-y

**Published:** 2024-04-09

**Authors:** Ashfaq Ahmad, Dong-You Choi

**Affiliations:** Information and Communication Engineering, Chosun University, Gwangju, 61452 South Korea

**Keywords:** Wide bandwidth, Metamaterial, mm-wave antenna, MIMO, Miniaturized, 5G, Electrical and electronic engineering, Nanoscale materials

## Abstract

In this paper, a wideband polarization conversion metasurface is designed. Additionally, coding and chessboard metasurfaces are specifically tailored for radar cross-section reduction (RCS). Initially, a compact unit cell demonstrating exceptional polarization conversion performance is introduced, achieving a polarization conversion ratio (PCR) exceeding 90$\%$ across frequencies ranging from 7.9 to 22.7 GHz. The PCR remains effective even when oblique incidence angles of up to 30^∘^ are utilized across this frequency band. Roger RT5880, with a thickness of 0.254 mm, serves as the substrate. An airgap is introduced between the substrate and the ground plane to enhance the polarization conversion bandwidth. This unit cell serves as the fundamental building block for subsequent metasurface configurations. To assess the scalability and effectiveness, a 36 $\times$ 36 unit array is assembled, confirming efficient polarization conversion capabilities extending to larger structures. Moreover, a 1-bit coding unit “0” and “1” are formed by the Pancharatnam-Berry phase based on the same-sized meta-atom with 90^∘^ orientations. The robustness and practicality of the design are demonstrated by creating 12 $\times$ 12 lattices and evaluating their RCS reduction potential under two distinct scenarios: a chessboard pattern and a coding-based scheme. Notably, its results indicate substantial RCS reduction across a broad frequency spectrum (7.9 to 22.7 GHz) for both configurations. This study demonstrates the wide-ranging applicability of metasurface design, making it a valuable contribution to the fields of microwave engineering, polarization control, and radar stealth technology. Owing to its simplicity, bandwidth, and versatility, this approach offers innovative solutions for diverse real-world applications.

## Introduction

The polarization of electromagnetic waves is a crucial aspect that requires attention. Controlling the polarization state has immense scientific significance for a wide range of practical applications. By enabling polarizers to enhance beam focusing, generate vortex beams, and power electromagnetic clocks, the ability to shape EM-wave polarization opens up a world of possibilities^1–4^. Traditional techniques for controlling electromagnetic polarization, such as the Faraday effect^5^ and birefringent crystals^6^, have long been at the forefront of this field of research. Despite their significance, these methods face several practical challenges, such as restricted bandwidth, large and cumbersome equipment, and difficulties in integration into modern systems. In recent years, metasurfaces, a type of two-dimensional metamaterial, have garnered significant attention. Their unique electromagnetic properties offer unparalleled opportunities for manipulation, making them of a special interest^7–10^. The advent of metasurfaces has paved the way for a new approach to controlling the characteristics of electromagnetic waves, including amplitude, phase, polarization, and propagation modes^11–14^. In contrast to conventional polarizers, metasurface-based polarizers have effectively addressed the limitations of traditional methods and showcased exceptional performance in the manipulation and application of electromagnetic waves^15–22^. This has captured widespread interest in academia and industry.

The two prevalent categories of polarization converters are transmission and reflection modes. For the transmission mode, a noteworthy approach involves the use of chiral metasurfaces, which have been proven to be remarkably effective in altering the polarization state of incident electromagnetic waves. They have demonstrated the ability to convert linear polarization into circular polarization^23^, circular-to-circular polarization^24^, and facilitate linear-to-linear^25^ transformations. However, there are certain practical constraints. These chiral metasurfaces tend to exhibit limited bandwidths^26^ and their intricate designs pose challenges for real-world applications. Reflective polarization converters are known for their advantageous energy efficiency compared with their transmission-based counterparts. Researchers have explored a variety of structures in this category, including the cut-rhombus shape^27^, 8-shaped^28^, L-shaped^29^, double-square-shaped^30^, Z-shaped^31^, and ellipse-shaped^32^, all of which have demonstrated the potential for achieving polarization conversion through reflection. However, it is important to recognize the challenges in this area. The pursuit of combining ultra-wideband capability with high efficiency, practical, and easy-to-use designs is a research area where researchers strive to make significant progress.

In addition to polarization conversion, another critical aspect of military technology is the reduction of the radar cross-section (RCS). This is becoming increasingly important owing to remarkable advancements in radar detection technologies. As these radar systems continue to evolve, the challenge of remaining undetected by radar becomes increasingly complex^33^. There are mainly two approaches to achieve low RCS using artificial structures^9^ The first approach involves using radar-absorbing materials, which absorb the energy of incoming waves, such as Salisbury screens^34^, circuit analog absorbers^35^, and frequency selective surfaces absorbers^36^. However, absorbing electromagnetic (EM) waves leads to a rise in temperature and increases the risk of infrared detection. Additionally, the thickness and structure of these absorbers limit their absorption bandwidth. Another approach for minimizing RCS is to scatter the reflected EM waves away from the source using diffusion metasurface^37^ In this regard, the coding metasurface was put forward by Cui et al.^38^ which introduced a novel concept for metasurface structure design, the unit cell with a constant phase difference at a desired frequency band can scatter electromagnetic waves irregularly to all direction, resulting in attenuation of echo signals and reduction of RCS. To achieve varying reflection phases one option is to use a similar unit cells with different dimensions, which cause bandwidth limitation and bring complexities to the design. The desired phase difference is achieved by rotating the anisotropic structure instead of employing multiple isotropic elements or adjusting specific size parameters, which is called the Pancharatnam-Berry (PB) phase. Several articles have utilized the aforementioned concept to minimize RCS^39^.In contrast to the existing literature, which often achieves limited or specific frequency bandwidth, and typically presents a single metasurface configuration, our proposed work introduces a novel approach by using wideband metasurface and demonstrate its effectiveness in reducing RCS using both chessboard and coding configurations.

The remainder of this paper is organized as follows. The outline begins in Section “Methodology”, where the methodology of the proposed unit cell is expounded, providing a detailed exploration of its working principle and performance. Section “Array configuration for polarization conversion” describes the array configuration tailored for polarization conversion and elaborates on its specifics. In Section “Coding and chessboard metasurface configuration for RCS reduction”, we discuss coding and chessboard metasurface and elucidate their roles in radar cross-section reduction. In Section “Comparison”, the present analysis is compared with existing state-of-the-art research. Finally, the last part concludes the paper by offering a conclusive summary and highlighting the key findings and contributions of this study.

## Methodology

### Unit cell configuration

**Figure 1:**
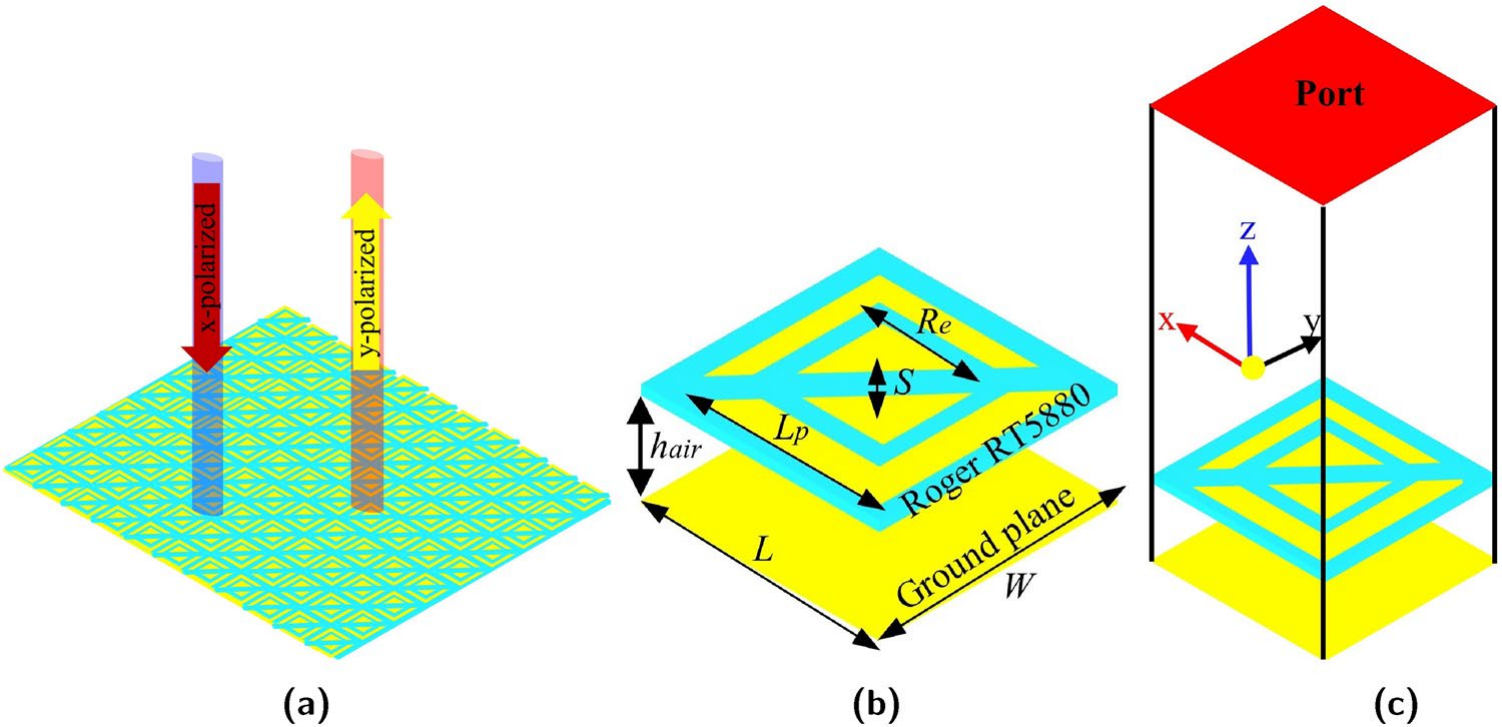
(**a**) Schematic diagram of the linear polarization converter (**b**) side view of a meta-atom (**c**) Three-dimensional view of the simulation setup.

**Figure 2:**
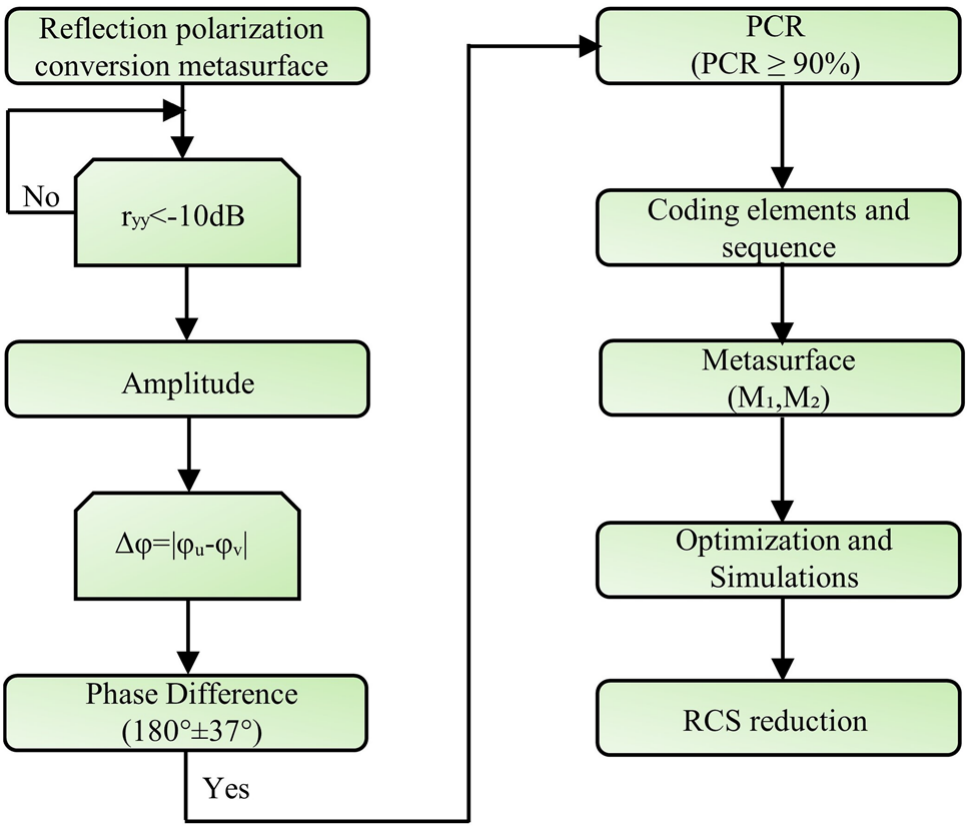
Design methodology flow chart of the PCR metasurface and coding bit metasurface to realize RCS reduction.

Figure 1 illustrates a schematic of the proposed linear-polarization converter, which contains three parts: the top patch is composed of a rectangular patch with diagonal and rectangular slots, an intermediate dielectric layer, and a bottom metal floor. Copper was selected as the metallic material with a conductivity of $5.8\times 10 ^{7}$ S/m and a thickness of 0.035 mm. Roger RT5880 was used as a substrate with a thickness of h = 0.254 mm, tangent loss of tan = 0.0009, and relative permittivity of 2.2. The patch size was fine-tuned using the HFSS software. The geometric parameters of the unit cell are shown in Fig. 1b, where S= 1.5, Re = 6.5, h = 0.254, h*a*ir = 2.8, and L = W = 9.5 mm. An air gap was added between the ground and substrate to increase the operating bandwidth^40^, this air gap serves as a substrate with low permittivity, the thickness of the air gap (h*a*ir) is optimized to achieve the highest bandwidth by merging the resonance frequencies, and a floquet port was used to excite the proposed unit cell. The x- and y-plane boundary conditions were set as unit cells, and an open (add space) boundary was applied in the z-direction. The z-axis represents the plane of incidence of the electromagnetic waves. The aim of this work was to design an ultra-wideband linear polarization conversion and RCS reduction using PB coding with a specifically arranged coding sequence. Figure 2. provides an overview of the design of a metasurface that converts polarizations and uses 1-bit Pancharatnam-Berry (PB) coding to significantly reduce the radar cross-section (RCS) over a wide bandwidth. Our design process starts by ensuring that the reflected waves have the correct amplitude and phase difference. Achieving a 180±37^∘^ phase difference is critical because it permits wide-band RCS reduction and excellent polarization conversion. Also, the polarization-conversion metasurface should be highly efficient in changing the polarization of incoming waves. Finally, simulations and optimizations were conducted to ensure that the metasurface performs well and analyze our goal of maintaining a low RCS across an ultra-wideband frequency band.

### Working principle and performance

**Figure 3:**
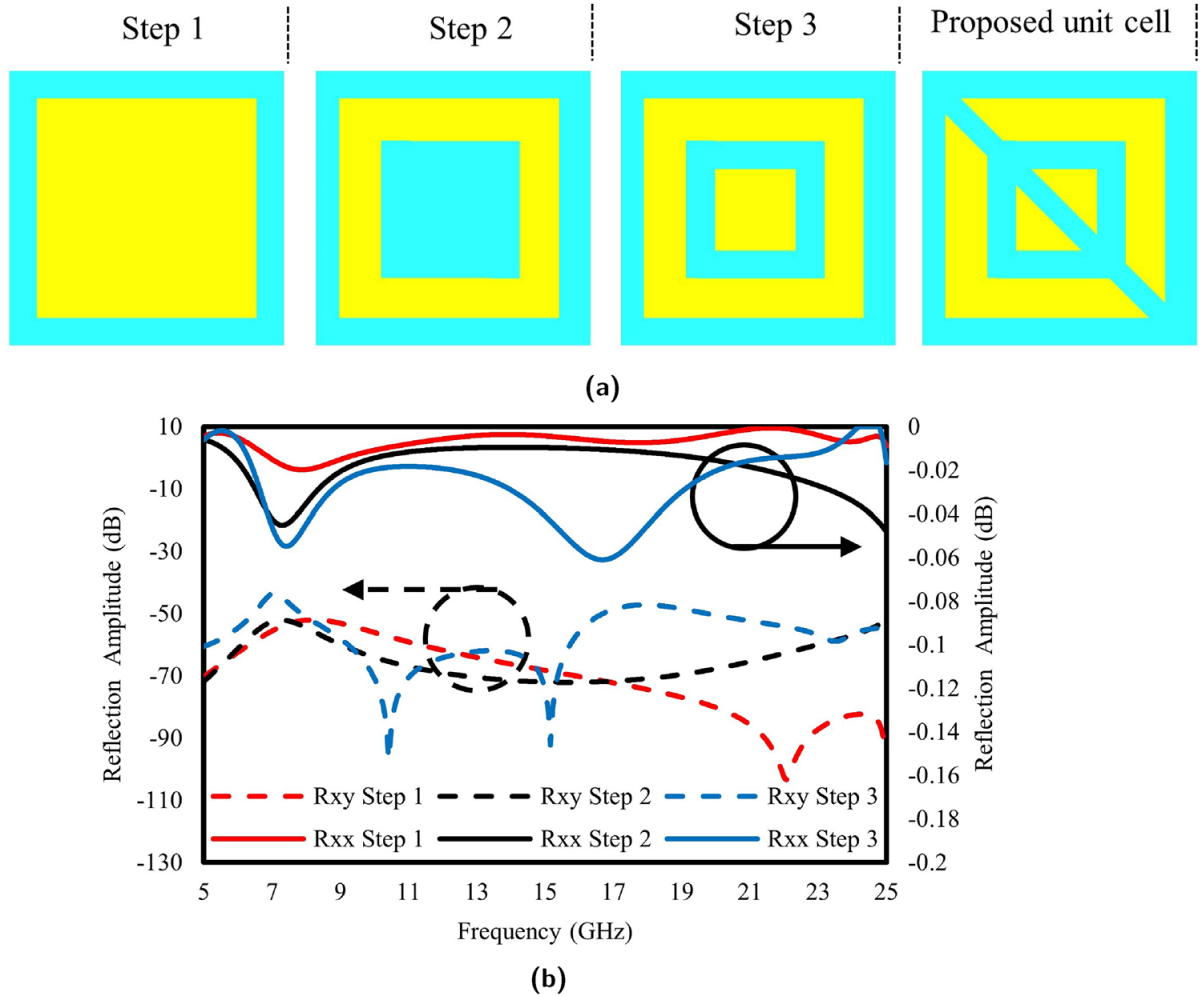
(**a**) Unit cell process (**b**) S-parameters of design process.

The proposed unit cell is designed in four steps. Initially, a simple patch structure, named Step 1, was designed, as shown in Fig. 3a. Subsequently, a rectangular slot was introduced, designated as Step 2, resulting in the observation of a single peak for each. However, upon introducing an additional small rectangular patch within the center of the slotted patch, two distinct resonances were detected at 7 GHz and 16 GHz, as shown in Fig. 3b. Finally, a diagonal slot was introduced into the radiating patches to achieve the desired results.

**Figure 4:**
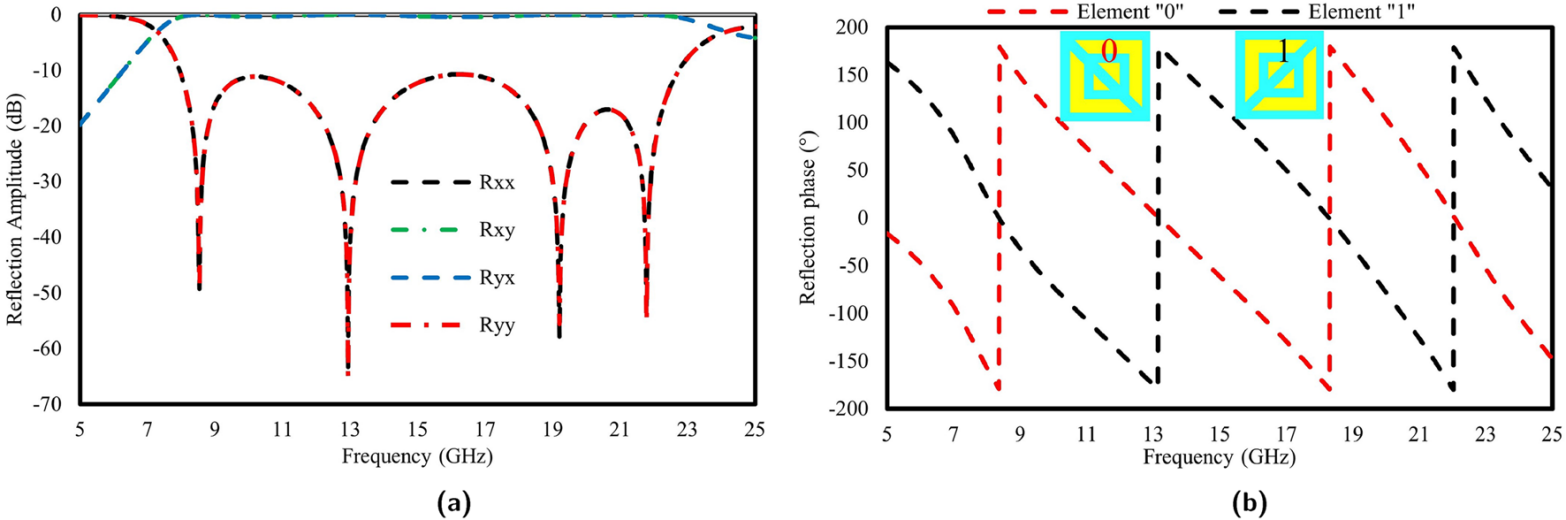
(**a**) Reflection Amplitude Characteristics of Meta-Atom ’0’ (**b**) Phase Analysis of Cross-Polarization Reflection between Meta-Atoms ’0’ and ’1’.

Figure 4a shows the reflected amplitude of the reflected meta-atom for linearly polarized incident waves. The cross-polarization reflection coefficient, characterized by Rxy and Ryx, demonstrates values exceeding -1 dB, while the co-polarized reflection coefficient, denoted by Rxx and Ryy, remains below -10 dB, as observed. with a wide frequency range of 7.9 GHz to 22.7 GHz. By utilizing the Pancharatnam-Berry (PB) phase, the unit “1” is formed, simplifying the overall structure. Based on this approach, the top metallic design can be rotated to generate different phase distributions. Rotating the top pattern by $\theta$ resulted in a phase shift of 2$\theta$. Considering this $\theta$ is rotated by 90^∘^ as shown in Fig. 4b, to provide a phase difference of ±180^∘^ for a 1-bit coding metasurface.

**Figure 5:**
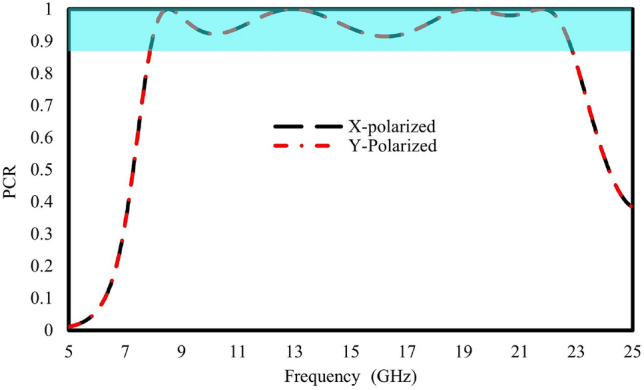
Polarization conversion ratio observed with incident x- and y-polarized waves.

To examine the polarization conversion of the designed meta-atoms, The polarization conversion ratio (PCR), which is computed for linearly polarized incident waves, describes the polarization conversion ability and can be calculated as

1 $$\begin{aligned} PCR_x=\frac{|R_{yx}|^2}{|R_{yx}|^2+|R_{xx}|^2} \quad \text {and} \quad PCR_y=\frac{|R_{xy}|^2}{|R_{xy}|^2+|R_{yy}|^2} \end{aligned}$$

where $PCR_x$ represents the polarization conversion efficiency of the x-polarized incident waves, and $PCR_y$ represents that of the y-polarized waves. The polarization capabilities of the meta-atoms are shown in Fig. 5. The illustration demonstrates a broad frequency range spanning from 7.9 GHz to 22.7 GHz, maintaining a polarization conversion exceeding 90$\%$ throughout the entire bandwidth. while for the four resonances ie: 8.5, 12.8, 19.03, and 21.88 GHz they reach 100$\%$. This indicates that the suggested meta-atom exhibits notable efficiency in polarization conversion, demonstrating its ability to effectively transform polarizations.

**Figure 6:**
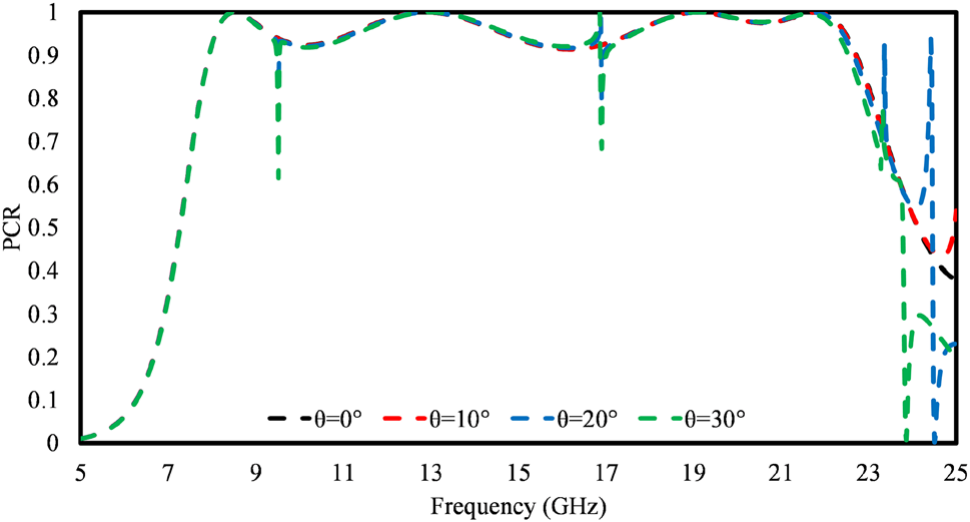
Polarization conversion ratio at various oblique incidences.

The incident angle stability is an important factor to consider while designing a metasurface for practical applications, where electromagnetic waves (EM) interact with the surface at different angles. Ideally, metasurfaces should exhibit consistent responses regardless of the incident angles of the impinging EM waves. For this, the unit cell is analyzed under different incident angles. The PCR efficiency of a unit cell under oblique incident EM waves is analyzed, as shown in Fig. 6. It is observed that the PCR efficiency remains above 90% across all operating ranges. However, small spikes are observed at 10 and 17 GHz for a $30^\circ$ incidence angle.

**Figure 7:**
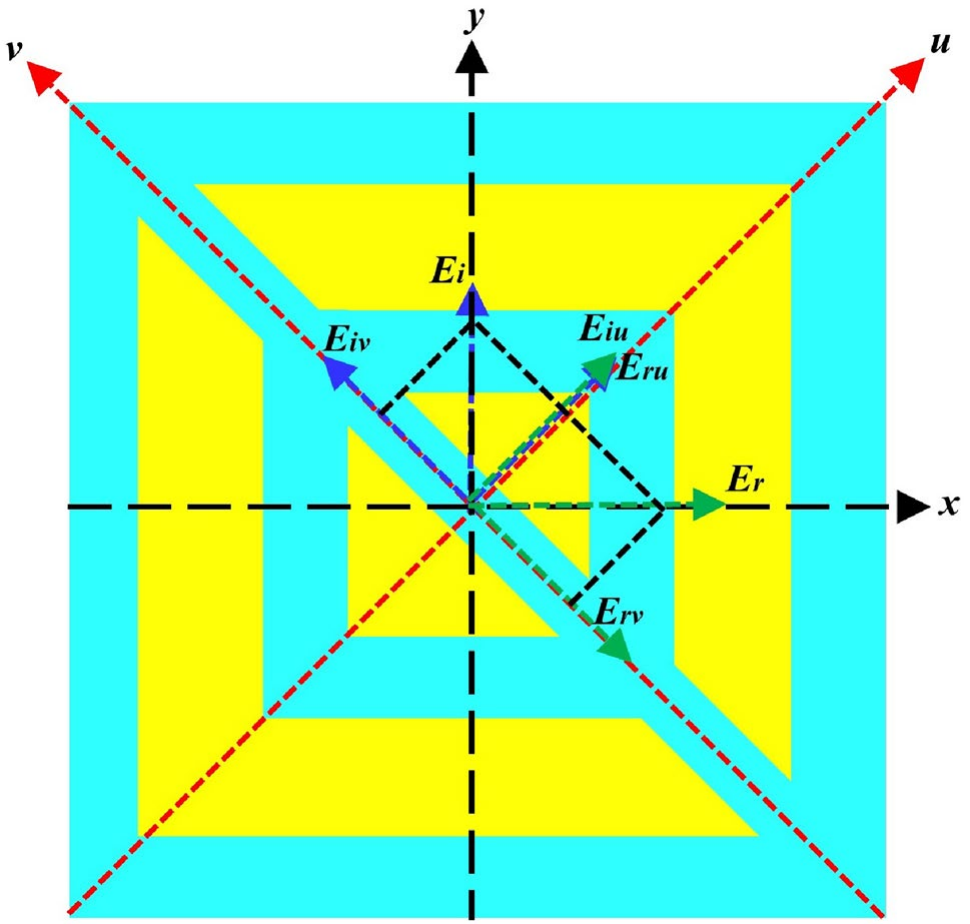
Visual representation of the operational principle through a schematic diagram.

For a more comprehensive analysis of the polarization conversion performance of the designed meta-atom, the design was simulated using the finite element method (FEM). We introduced the u- and v-axes, derived by rotating the x- and y-axes by 45^∘^ anticlockwise, as depicted in Fig. 7. The equations describing the incident and reflected waves are as follows:

2 $$\begin{aligned} E_i=yE_i=uE_{iu}+vE_{iv} \end{aligned}$$

3 $$\begin{aligned} E_r=uE_{ru}+vE_{rv}=ur_uE_{iu}+vr_vE_{iv} \end{aligned}$$

The unit vectors are denoted by *u* and *v*, and the reflection coefficients along the *u* and *v*-axes are denoted by $r_u$ and $r_v$, respectively. Equation (3) can be simplified as^41^.

4 $$\begin{aligned} E_r=u(r_{uu}E_{iu}e^{i\phi uu}+r_{uv}E_{iv}e^{i\phi uv})+v(r_{vv}E_{iv}e^{i\phi vv}+r_{vu}E_{iu}e^{i\phi vu}) \end{aligned}$$

In Eq. (4), $r_{uu}$, $r_{vv}$, $r_{uv}$, and $r_{vu}$ are the co- and cross-coefficient magnitudes, and the phases are given as $\phi _{uu}$,$\phi _{vv}$, $\phi _{uv}$, and $\phi _{vu}$ along the u- and v-axes, respectively.

**Figure 8:**
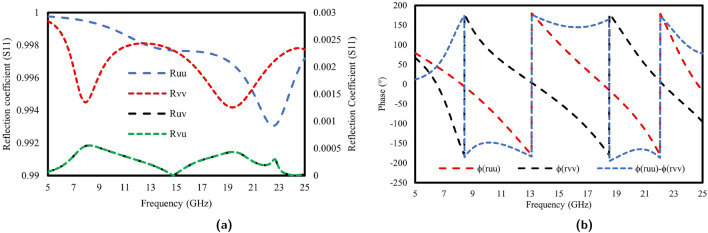
(**a**) Magnitude and (**b**) Phase of the reflection coefficient of *u* and *v*-waves.

Figure 8a and b, present the phase response and magnitude of the reflection coefficients for the u- and v-waves at different frequencies and evaluate the behavior of the designed meta-atom. Specifically, Fig. 8a shows the amplitudes of the co-polarized components, |$R_{uu}$| and |$R_{vv}$|, along with the cross-polarized components, |$R_{uv}$| and |$R_{vu}$|, in reflection. Notably, within the desired frequency range, |$R_{uu}$| and |$R_{vv}$| exhibit values that exceed 0.9, whereas |$R_{uv}$| and |$R_{vu}$| remained at nearly zero, primarily because of the anisotropy of the proposed meta-atom. This results in a phase difference $\Delta \phi$ between the u- and v-polarized waves. As shown in Fig. 8b, the phase disparity between the u- and v-reflected waves consistently approaches approximately ±180^∘^ across the entire frequency spectrum. This observation indicates that the designed converter is capable of performing linear cross-polarization conversion consistently over the whole frequency range^42^.

**Figure 9:**
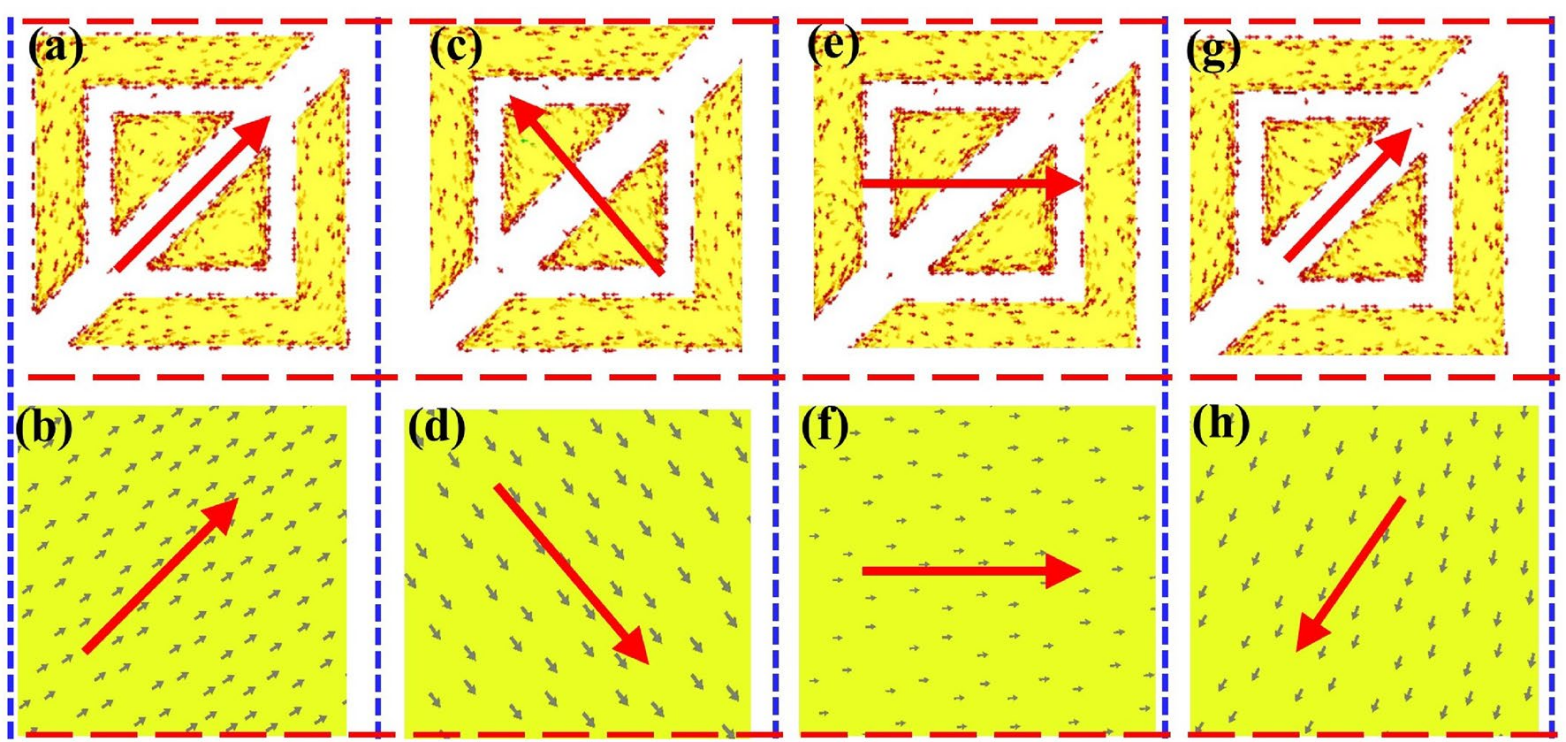
Surface current distribution on the top layer and metallic ground plane at different resonant frequencies. (**a**, **b**) 8.5 GHz. (**c**, **d**) 12.8 GHz. (**e**, **f**) 19.03 GHz. (**g**, **h**) 21.88 GHz.

Figure 9a–h shows the surface current distribution, providing a visual representation of the polarization conversion phenomenon. The surface current distributions observed at various resonance frequencies (8.5, 12.8, 19.03, and 21.88 GHz) on the top and bottom layers signify the electric or magnetic resonance of the proposed unit cell. Electrical resonance occurs when surface currents align in parallel on both the top patch and the lower metallic floor, and magnetic resonance occurs when currents on these layers flow in opposite directions. The surface currents on the metallic patch oppose those on the ground plane at frequencies of 12.8 and 21.88 GHz, indicating magnetic resonance. Conversely, at frequencies 8.5 and 19.03 GHz, the current distributions on both layers align, indicating electric resonance. As reported in^43^, the robust electromagnetic resonance is attributed to the effective polarization conversion achieved by the metasurface within the desired frequency bands.

**Figure 10:**
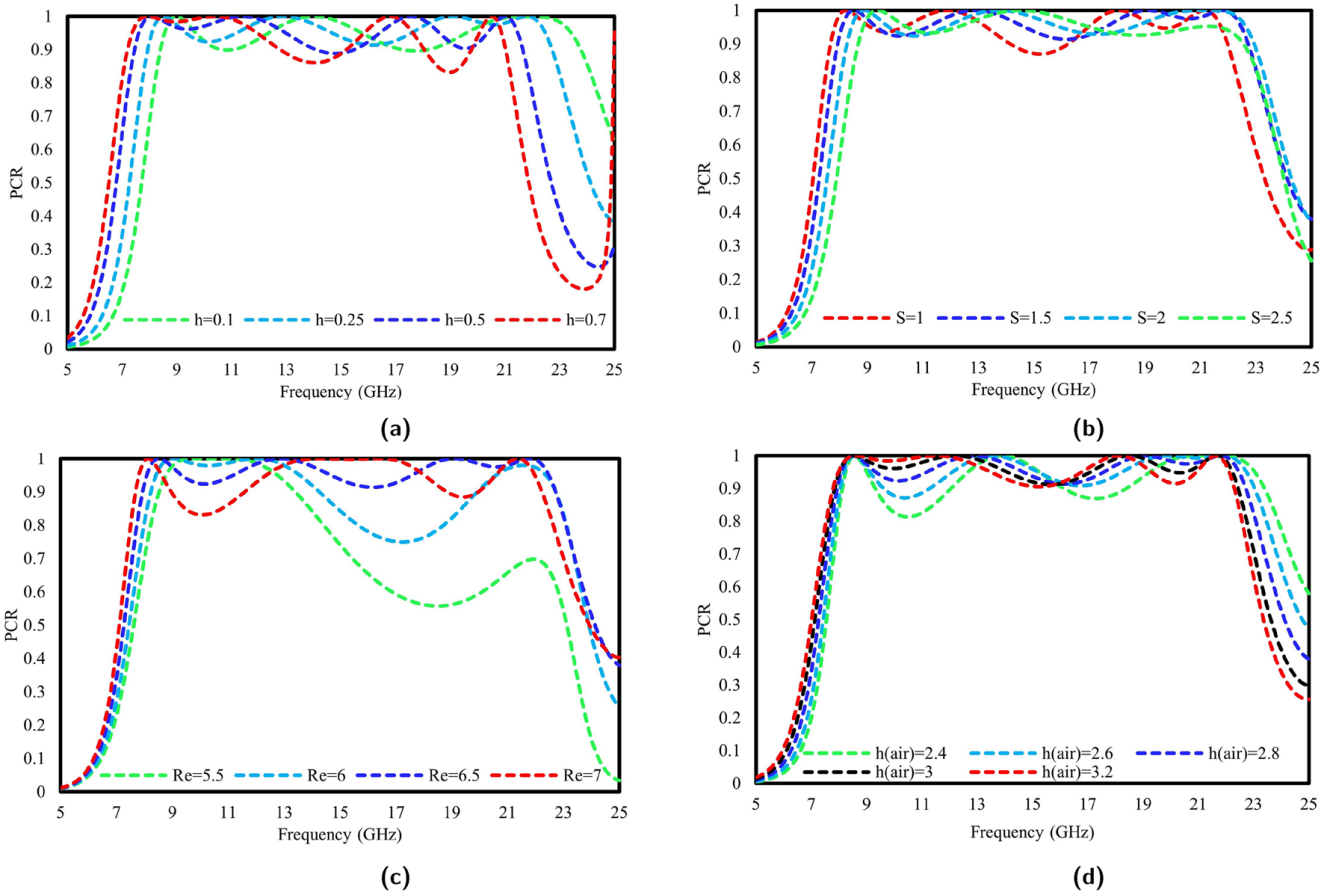
Modifications in polarization conversion ratio with alterations in: (**a**) substrate thickness, (**b**) diagonal strip width, (**c**) inner patch size, and (**d**) air gap.

The PCR underwent an additional analysis to assess the impact of the geometric attributes on the performance through a parametric analysis. This analysis enables the identification of an optimized structure, as illustrated in Fig. 1. The key parameters that influence the performance include the substrate height (h), width of the diagonal strip (S), size of the inner patch (Re), and air gap (h*air*). The impact of the substrate’s thickness is shown in Fig. 10a.; the frequency band shifts toward the lower side as the thickness of the substrate increases. Similarly, the width of the diagonal slot has a minimal effect on the performance of the unit cell. Its performance was analyzed from 1 mm to 2.5 mm, as shown in Fig. 10b. Another crucial factor for the proposed meta-atom is the dimensions of the inner patch. As observed from Fig. 10c, the PCR for various values of Re is analyzed from 5.5 mm to 7 mm. For the small size of the inner patch, i.e., Re= 5.5 mm, the PCR values are observed to be extremely low, much less than 60$\%$ for the higher frequency band. Among them, the best value of 6.5 mm was selected for our design unit cell, as it shows a PCR of more than 90$\%$ for the entire frequency band. In Fig. 10d, the variation in the polarization conversion is observed by changing the distance between the substrate and the ground plane.

## Array configuration for polarization conversion

**Figure 11:**
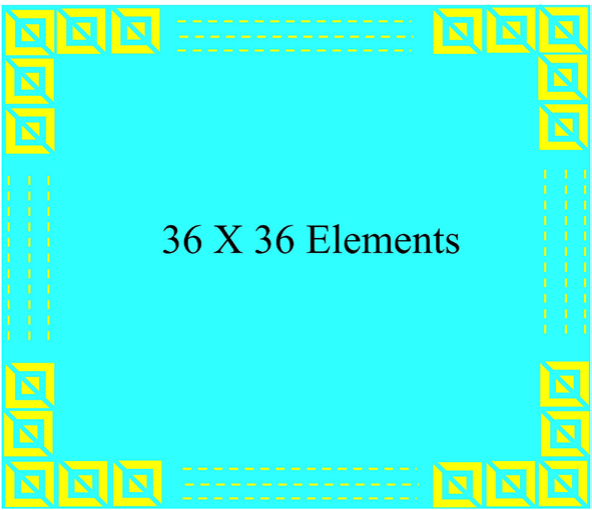
36$\times$36 units polarization conversion metasurface.

**Figure 12:**
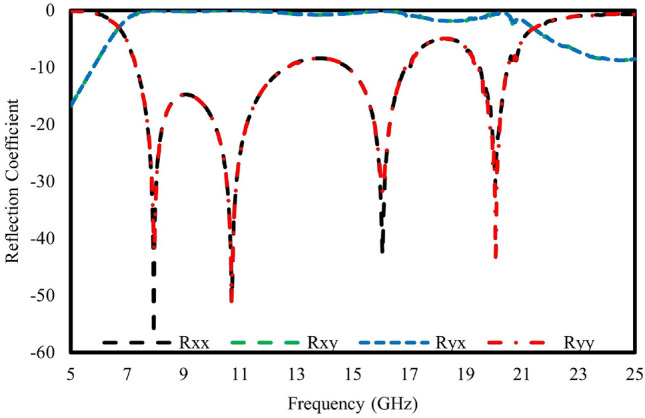
S-parameter of the polarization conversion metasurface.

Figure 11 illustrates a 36 $\times$ 36 elements array of the proposed unit cell to validate its polarization conversion property. Figure 12 shows the amplitude responses of the reflected metasurface exposed to incident waves with linear polarization. Notably, the reflected co-polarization coefficients, Rxx and Ryy, exhibit levels below -10 dB across the frequency range from 7.9 GHz to 22.7 GHz. Conversely, the cross-polarization coefficients, Rxy and Ryx, demonstrate values exceeding -1 dB over the same frequency range; however there is a small varions observed at the higher frequency range.

**Figure 13:**
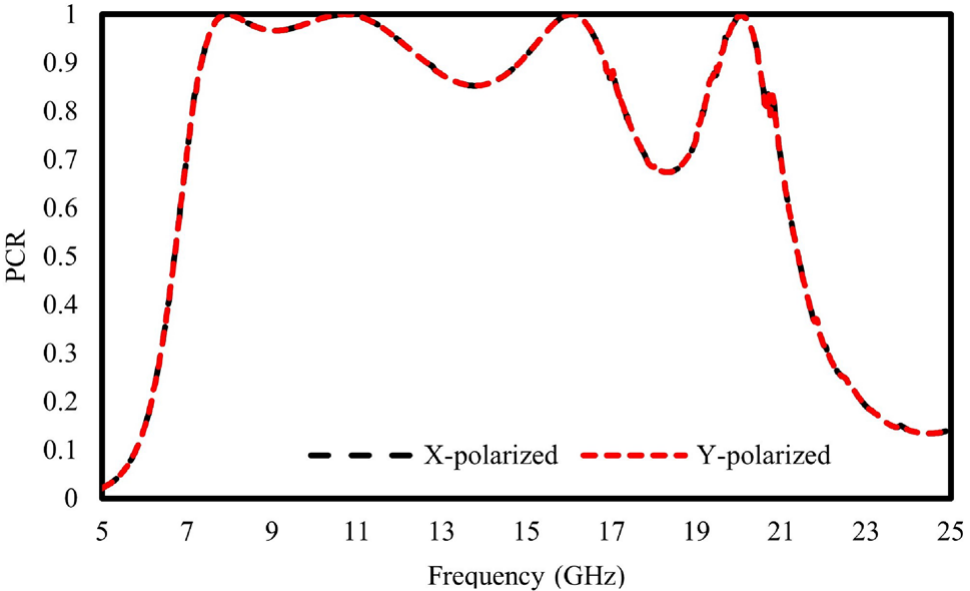
PCR of 36$\times$36 units metasurface.

Figure 13 shows the PCR of the designed 36$\times$36 unit metasurface. It is observed that the metasurface demonstrates a PCR exceeding 90% within the frequency range of 7.9 GHz to 12 GHz. Subsequently, the PCR declines to approximately 80% between 12 GHz and 16 GHz. Notably, another decline in PCR occurred within the 17 GHz to 19.5 GHz range, reaching a minimum of approximately 68% at 18.8 GHz. Overall, it can be deduced that the proposed metasurface exhibits excellent PCR performance across the entire frequency band.

## Coding and chessboard metasurface configuration for RCS reduction

To assess Radar cross-section (RCS) performance, A 1-bit coding metasurface comprising polarization conversion meta-atoms was employed to assess the RCS performance. This metasurface is structured with lattices, where each lattice comprises alternating “0” and “1” meta-atoms, each assigned a scattering phase of either 0 or $\pi$. A coding metasurface is created by the distribution of different coding elements with varying phases while the amplitude remains the same. With the introduction of the PB phase, an anisotropic structure can be rotated easily to achieve the phase difference. According to^39^ the far-field scattering by coding metasurface can be expressed as

5 $$\begin{aligned} f(\theta ,\varphi )=f_{m,n}(\theta ,\varphi ).AF(\theta ,\varphi ) \end{aligned}$$

6 $$\begin{aligned} AF(\theta ,\varphi )=\sum _{m=1}^{M}\sum _{n=1}^{N}exp \left\{ {{{ j \varphi (m,n)}}+ kDsin\theta \times {[(m-1/2) cos\varphi + (n-1/2)sin\varphi }}] \right\} \end{aligned}$$

Where $f_m,_n (\theta ,\varphi )$ and $AF (\theta ,\varphi )$ indicate the radiation intensity of each element and the array factor, respectively. *k* is the wavenumber vector and *D* is the size of the lattice. $\theta$ is the elevation angle, and $\varphi$ is the azimuth angle. the scattering phase of each lattice is assumed to be $\varphi (m,n)$, which is either $0^\circ$ or $180^\circ$, therefore, $f_m,_n (\theta ,\varphi )$ is removed due to the phase difference between ’0’ and ’1’ and the directivity function $Dir(\theta ,\varphi )$ of the metasurface is expressed as follows:

7 $$\begin{aligned} D(\theta ,\varphi )= \frac{4\pi \left| f(\theta ,\varphi ) \right| ^2 }{\int _{0}^{2\pi }\int _{0}^{\pi /2} \left| f(\theta ,\varphi ) \right| ^{2} sin\theta d\theta d\varphi } \end{aligned}$$

Relative to a metallic plate with the same size the RCS reduction caused by the coding metasurface is obtained as:

8 $$\begin{aligned} RCS_{Reduction}= \frac{\lambda ^{2} }{4\pi N^{2} D^{2}}Max\left[ D(\theta ,\varphi ) \right] \end{aligned}$$

**Figure 14:**
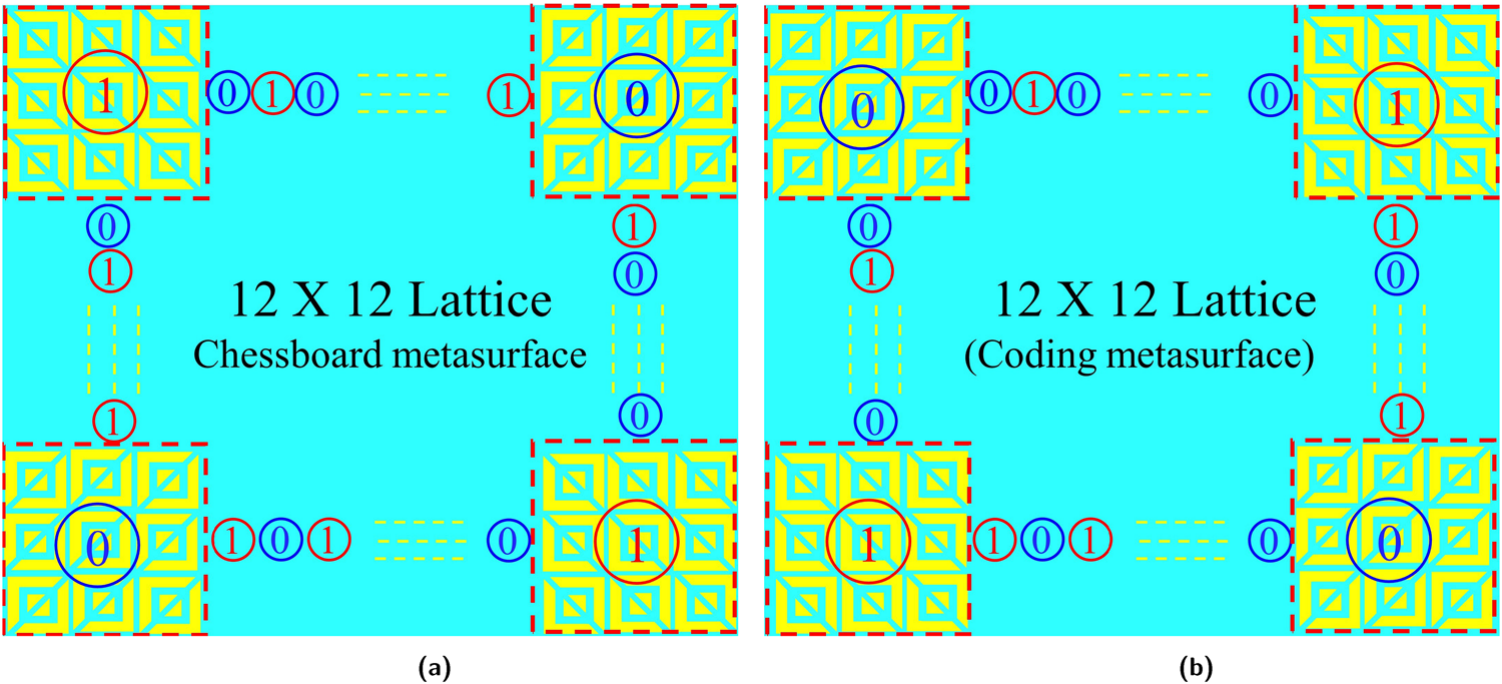
Schematic diagram of (**a**) chessboard metasurface (**b**) coding metasurface.

From the above Eqs. (7) to (8), we observe control over the scattered field through the coding metasurface elements. The sequence “001001110101” represents the optimized arrangement of these lattices, enabling a significant RCS reduction surpassing 10 dB when the phase difference between them approximates $\pi$. The general configuration of the optimized chessboard metasurface is depicted in Fig. 14a. In this depiction, the red color symbolizes lattice “1,” while the blue color signifies lattice “0.” Furthermore, as shown in Fig. 14b, the metasurface containing the coding sequences was examined to verify the design scheme’s wide applicability.

**Figure 15:**
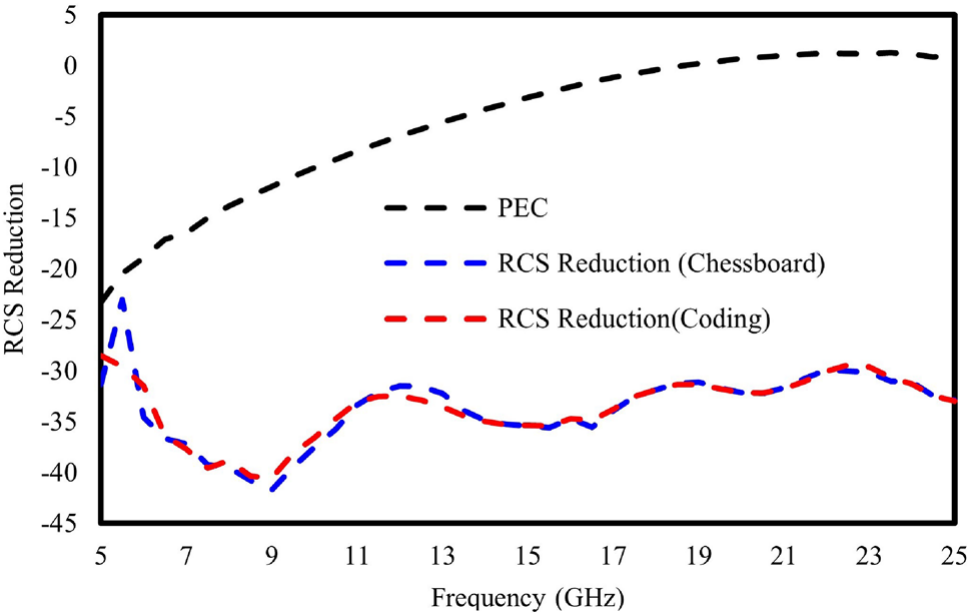
RCS reduction for chessboard and coding metasurface.

Figure 15 presents an insightful comparison through RCS reduction plots between two distinct metasurfaces: the chessboard metasurface and coding sequence metasurface. Additionally, RCS reduction plots for structures made of perfect electric conductors (PEC) of equivalent size are depicted. Both the chessboard and coding sequence metasurfaces exhibited consistent and noteworthy RCS reduction capabilities, showing reductions exceeding 10 dB across the entire frequency range under investigation. This substantial reduction in RCS is noticeably superior to that achieved by PEC structures with equivalent dimensions.

**Figure 16:**
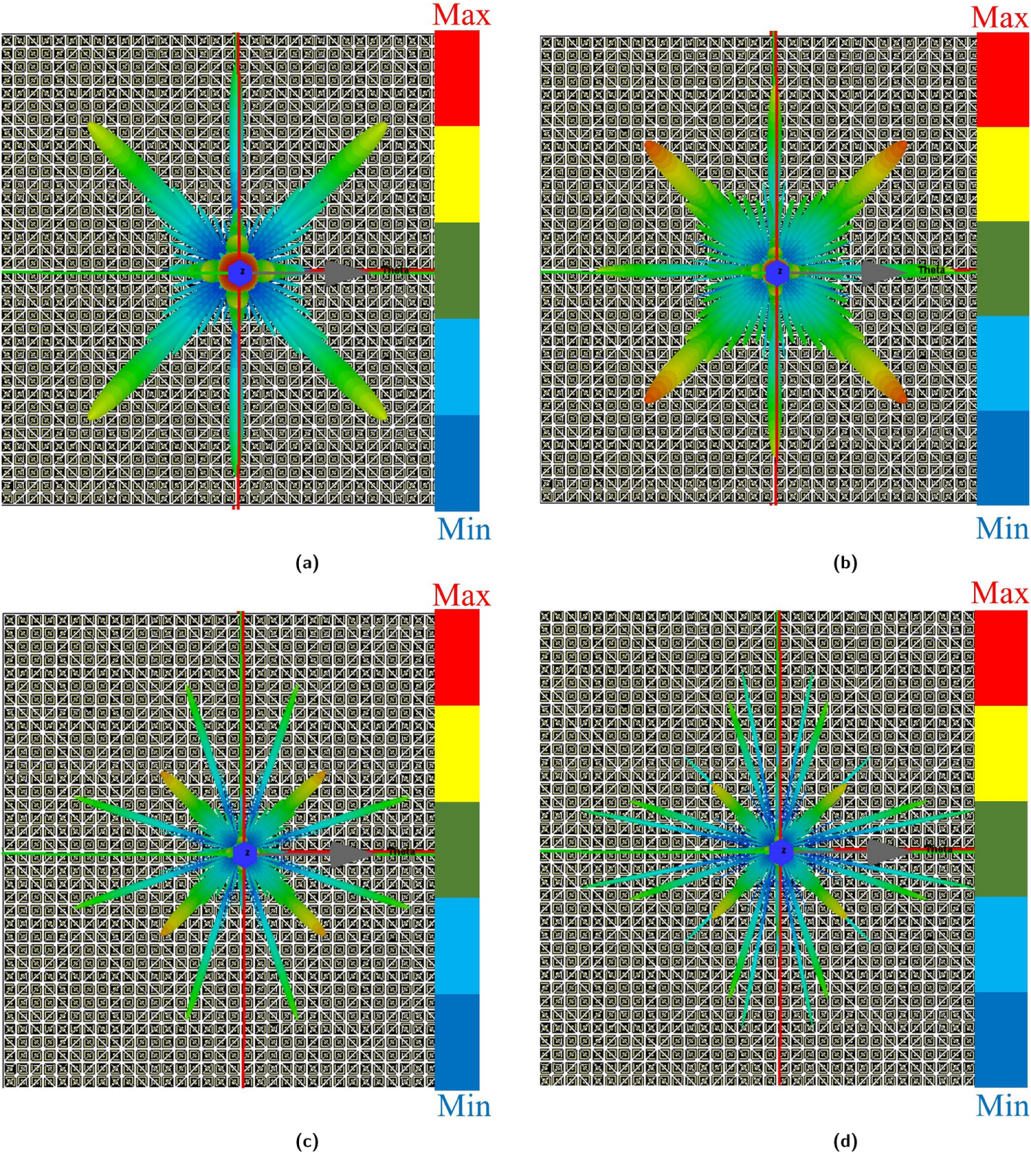
3D radiation pattern of chessboard metasurface (**a**) 8.5 GHz, (**b**) 12.8 GHz, (**c**) 19.03 GHz, (**d**) 21.88 GHz.

To provide additional insight into the chessboard metasurface’s operation, The 3-D patterns under normal incident waves at 8.5, 12.8, 19.03, and 21.88 GHz, respectively, are shown in Fig. 16a–d. Discerning these figures reveals the scattering of the reflected waves in multiple directions, thereby effectively rendering the surface less detectable.

**Figure 17:**
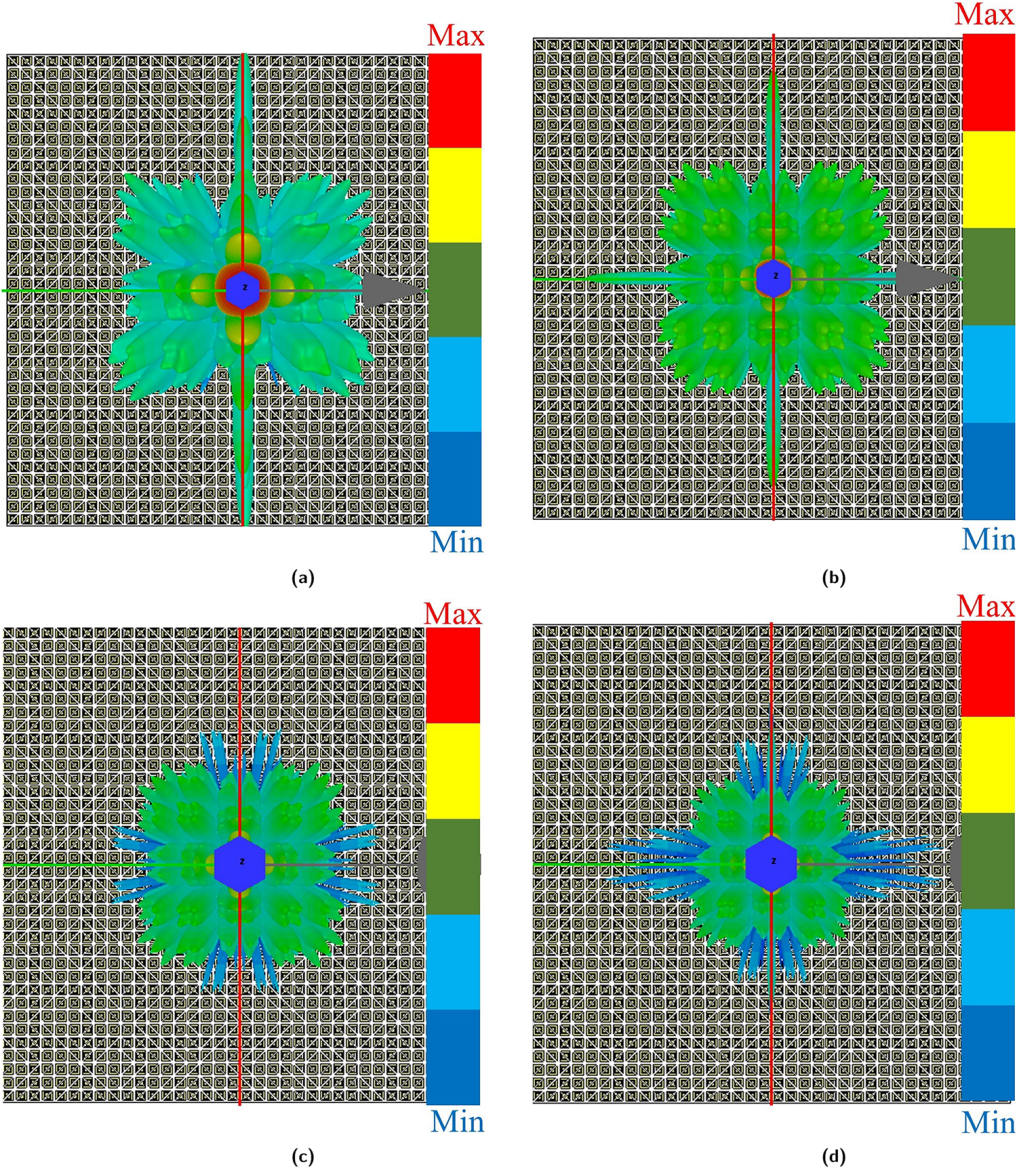
3D radiation pattern of coding metasurface (**a**) 8.5 GHz (**b**) 12.8 GHz (**c**) 19.03 GHz (**d**) 21.88 GHz.

Similarly, Fig. 17a–d illustrates the 3-D patterns under normal incident waves at 8.5, 12.8, 19.03, and 21.88 GHz, respectively. There was a significant reduction in the backward RCS reduction as a result of the reflected waves being uniformly distributed in all directions.

## Comparison

Table 1 presents a comparison between the current study and the recently published state-of-the-art research. The comparison encompasses aspects such as the frequency range, fractional bandwidth, methodology employed for RCS reduction, and polarization conversion ratio.

**Table 1:** Comparison table.

**Ref.**	Unit cell size (mm)	Frequency range (GHz)	Fractional bandwidth (%)	RCS reduction mechanism	PCR
^8^	$6 \times 6$	10.8–31.3	102	Absorption + diffusion	>90%
^44^	$9 \times 9$	11.5–16	32	Absorption + diffusion	–
^45^	$7 \times 7$	5.4–9, 14.6–16	50, 9.8	–	93–99%
^46^	$15\times 15$	4.2–11.6	93	Diffusion	–
^47^	$10\times 10$	9.5–14, 15.2–20.4	32, 30	Diffusion	98, 99
Proposed work	$9 \times 9$	7.9–22.7	96.7	Absorption + diffusion	>98

## Conclusion

This study introduced a novel polarization-independent coding metasurface that achieves a broad reduction in the radar cross-section (RCS) for polarization conversion, diffusion, and absorption (PCDA) mechanisms. To assess the scalability and effectiveness, a $36 \times 36$ unit array is assembled, confirming efficient polarization conversion capabilities extending to larger structures. Similarly, coding and chessboard metasurfaces are specifically tailored for RCS reduction. The unit cell “0” comprised a slotted rectangular structure coupled with a diagonal strip, while the utilization of the PB phase enables the creation of meta-atom “1” forming a 1-bit coding metasurface. Through the tailored meta-atom arrangement, this coding metasurface effectively minimized backscatter across a vast frequency spectrum by employing diffusion and absorption concurrently. Notably, this metasurface exhibited a reflection reduction of more than 10 dB across azimuthal angles ranging from 7.9 to 22.7 GHz. The integration of PCDA mechanisms along with robust angular and polarization stabilities highlights the significant potential of this approach for advancing stealth EM technologies.

## Data Availability

All the data generated or analyzed during this study are included in this published article.
